# Ignored Disease or Diagnostic Dustbin? Sudden Infant Death Syndrome in the British Context

**DOI:** 10.1093/shm/hkv003

**Published:** 2015-03-02

**Authors:** Angus H. Ferguson

**Keywords:** SIDS, cot death, diagnosis, medicalisation, symptoms, frameworks of disease

## Abstract

Sudden Infant Death Syndrome (SIDS) was defined in 1969 and incorporated into the International Classification of Diseases a decade later. To advocates of SIDS as a diagnosis, medical interest in sudden infant death was long overdue. However, the definition of SIDS lacked positive diagnostic criteria, provoking some to view it as a ‘diagnostic dustbin’ for the disposal of problematic cases where cause of death was unclear. This paper examines the development of medical interest in sudden infant death in Britain during the middle decades of the twentieth century. It highlights the importance of recognising the historicity of SIDS as a diagnosis facilitated by changes in law and medicine over the course of the nineteenth and twentieth centuries. It suggests that SIDS provides a definitive case study of the medicalisation of life and death, and a unique example of an officially recognised disease that had no symptoms, signs, pathology or patients.

The concept of a disease is thus an abstraction from the reality of phenomena observed in patients, useful because it permits of thinking, speaking, and writing in generalisations.^1^

Sudden Infant Death Syndrome (SIDS) was defined and adopted as a diagnosis during the second international conference on the causes of sudden death in infants, held in Eastsound, Washington in 1969.^2^ The inaugural conference, designed to review recent work and recommend future research priorities, had taken place in Seattle in 1963. Abraham Bergman, an American paediatrician and central figure in promoting medical interest in sudden infant death, noted that at the time of the first conference ‘the biggest unknown was whether the bulk of infants dying suddenly and unexpectedly were victims of a distinct disease entity or were dying coincidentally from a number of known diseases’.^3^ By 1969, according to Bergman, the picture was becoming a great deal clearer.^4^ In 1978, the ninth revision of the International Classification of Diseases added SIDS to the list of officially recognised diseases.^5^

The attention SIDS has received in recent decades is often portrayed as a stark contrast to ignorance of it in earlier periods. In recounting his experience of championing SIDS as a disease requiring focused research by medical science, Bergman asserted that ‘despite the magnitude of the problem, until the mid 1970s SIDS must have been the most ignored disease in history.’^6^ However, SIDS has often proven to be controversial—not least because it is a diagnosis of exclusion. The 1969 conference defined SIDS as ‘the sudden death of any infant or young child, which is unexpected by history, and in which a thorough post-mortem examination fails to demonstrate an adequate cause for death’.^7^ In other words, cases were not identified using positive criteria, such as an agreed pattern of symptoms, signs or explanatory pathology, but by the absence of evidence for an alternative cause of death.^8^ Consequently, SIDS was something of an anomaly in medicine: an officially recognised disease with no symptoms, no signs, no explanatory pathology and no patients. John Emery, Professor of Paediatric Pathology in Sheffield, noted that the recommendation that all such cases should be registered as Sudden Infant Death Syndrome was rapidly adopted internationally. He pointed to five key reasons for this:

It enabled doctors to tell parents that their child had died of natural causes and that no one could have prevented it. It excused all concerned from any defect in care, diagnosis and treatment. Pathologists welcomed the diagnosis: the less they found, the more certain they could be. Because the syndrome was of unknown origin health authorities had no basis for prevention. And finally, this diagnosis facilitated the development of parent support groups and the raising of money for research.^9^

However, the absence of positive diagnostic criteria for SIDS was a source of concern. Emery's article queried whether SIDS was a real disease or a ‘diagnostic dustbin’ for the disposal of cases in which investigation failed to reveal an obvious cause of death.^10^

This paper explores Bergman's assertion that SIDS was an ignored disease, and Emery's characterisation of it as a diagnostic dustbin, through the analysis of medical interest in sudden infant death in Britain during the years prior to the definition and recognition of SIDS in 1969. While sudden infant death became a focus of international medical research, there are good reasons to focus on Britain. In America the promotion of SIDS as a legitimate diagnosis was important in securing federal funding for research.^11^ In Britain, by contrast, both the Ministry of Health and the Medical Research Council (MRC) were active in funding research, combining investigation of possible clinical, pathological and social causes, prior to the definition of SIDS. Indeed, as detailed in later sections of the paper, by the mid-1960s the MRC was struggling to persuade researchers to apply for available funding. Therefore, while only part of the bigger picture of international work on SIDS, Britain presents an important case study of the lead up to the definition and recognition of SIDS.

The analysis contextualises the medicalisation of sudden infant deaths using the historiography of death registration, infant mortality and relevant changes in law and medicine in the nineteenth and early twentieth centuries, highlighting the importance of recognising the historicity of SIDS.^12^ Having discussed why medical interest came to focus on SIDS in the mid-twentieth century, the paper examines the early co-ordinated studies of sudden infant death in Britain, investigating the extent to which their findings contributed to the discovery of a new disease. The final section of the paper examines SIDS in relation to the social construction of disease and the frameworks of understanding used by historians of medicine. It concludes that, while the lack of positive diagnostic criteria entails that SIDS does not fit into traditional frameworks of disease, the diagnosis had power and significance. This was especially so for the parents and families of victims for whom a medical explanation, even one devoid of diagnostic, prophylactic or therapeutic substance, helped to assuage guilt and legal suspicion and facilitated the establishment of interdisciplinary support networks.

## Ignored Disease?

Civil registration of vital statistics was instituted in nineteenth-century Britain through legislation requiring the recording of births, marriages and deaths in England and Wales in 1836 and Scotland in 1854.^13^ Although the initial drive behind civil registration centred on facilitating the transfer of property to legitimate heirs, over time the use to which registration was put, and its relevance to medicine, grew. From the late nineteenth century, the dual emphasis on ensuring that certification of death was carried out by doctors and that it included a specific cause, framed in clinico-pathological terms, was vital to the rise of medical interest in SIDS.

While many analyses of SIDS begin by pointing to evidence of sudden infant deaths dating back to biblical times, there are reasons why coordinated medical interest focused on the issue in the middle decades of the twentieth century.^14^ In the UK, prior to the advent of civil registration of vital statistics, infants did not attract much medical or legal concern.^15^ Therefore sudden infant deaths, specifically, were not ignored, rather they were a subset of a broader group of deaths that received little attention. From 1877 onwards the annual report of the Registrar General included figures for infant mortality.^16^ This highlighted a problem of persistently high infant mortality, in contrast to a pattern of annually falling rates of mortality in the general population.^17^ As David Armstrong notes ‘the creation of a specific mortality rate for infants at this time suggests both the emergence of a social awareness of these young deaths and, more importantly, the social recognition of the infant as a discrete entity.’^18^

Official recognition of infants as a distinct group for analysis had significant legal implications. Recording the birth and death of infants was an important step towards legislation safeguarding such vulnerable lives. Evidence of an increasing concern about the fate of infants is found in the *Infant Life Protection Act*, 1897, which required that suspicious infant deaths be notified to district coroners, or, in Scotland, to the procurator fiscal, so that an inquest to establish the cause of death could be carried out.^19^ Under the terms of the *Children Act*, 1908, the overlaying of a child by an adult under the influence of drink was made a criminal act of negligence.^20^ Such legislation demonstrated a perceived need to protect infants from parental neglect or malevolence.^21^ As the predominant explanation given for unexpected infant deaths, ‘overlaying’ both reflected and perpetuated contemporary concerns about the working, drinking and sleeping habits of the working class population.^22^

Anne Carmichael and George Alter indicate that, at its outset, registration of vital statistics was motivated by the interest of statisticians in recording population trends and patterns rather than by medical theory or practice.^23^ However, as doctors became more involved in the process of registering cause of death, a tension arose between the interests of doctors and statisticians.

The emergent statisticians of the eighteenth and early nineteenth centuries were interested in statistical classification—that is, in grouping individual entities in such a way as to derive general principles governing deaths. The medical men were interested in nomenclature, in assessing distinctions with a view to understanding multiple causes of morbid phenomena. In other words, one group were lumpers, the other splitters.^24^

Both agendas had implications for SIDS. As already noted, the statisticians' categorisation of registration data highlighted infant mortality as a specific area of concern, which was a factor in sparking legal and medical interest. Paediatrics became established as a distinct specialism during the early decades of the twentieth century, with subdivisions focusing attention on perinatal, neonatal and postneonatal conditions.^25^ This period produced increased medical interest in stillbirths, with legislation requiring registration of stillbirths in England and Wales from 1927, and in Scotland—with the additional requirement that a specific cause be given—a decade later.^26^

On one level, there are similarities between the rise of medical interest in stillbirth and sudden infant death. Cases of either often attracted legal rather than medical attention, with suspicion of abortion or infanticide driving investigation. Similarly, once medical attention came to focus on recording and examining cases in more detail, both stillbirths and sudden infant deaths were found to be much more common than previously thought. In the middle decades of the twentieth century, stillbirths were recognised as the single largest cause of reproductive mortality.^27^ Early studies in the 1950s suggested that sudden infant deaths might account for around 20 per cent of postneonatal infant mortality, and changes in registration practices entailed that it rose to account for nearly half of postneonatal infant mortality in the 1970s and 1980s.^28^

However, the differences between stillbirth and SIDS are significant in terms of their respective medicalisation. Setting aside cases of abortion or infanticide, medical explanations of stillbirth focused on intrauterine conditions, particularly the age, health and nutrition of the mother.^29^ The mother, as much as the fetus, was the patient—arguably more so until ultrasound enabled the medical gaze to focus on the placenta in the 1960s—and the search for causal explanations considered both.^30^ By contrast, SIDS typically affected the postneonatal infant population, with the number of cases peaking around the age of 2–4 months, when the viability and separate existence of the infant was established. As Bergman noted, ‘SIDS does not claim the tiniest, most fragile, immature victims. It is not a phenomenon of the newborn period.’^31^ Reflecting this, early studies on sudden infant death generally defined cases for examination in a way that separated sudden infant death from stillbirth. For example, the UK Ministry of Health's study in the 1950s specified the age range of interest as ‘from two weeks to two years’ old.^32^ Other international studies focused on the period 2–11 months.^33^ While recent research has sought to establish a link between obstetric conditions and particular cases of sudden infant death, earlier medical research often linked the latter with examples of sudden death in childhood or adulthood.^34^ As discussed below, early attempts to identify, diagnose and avert potential cases of sudden infant death had devastating consequences for healthy infants.

Alter and Carmichael note that doctors' growing involvement in death registration ‘created a set of relationships between physicians and patients that was not linked to medical care and did not necessarily reinforce the physician's role as comforter or healer’.^35^ This was an important step towards SIDS, an exclusively retrospective and non-therapeutic diagnosis, becoming the focus of medical interest. Moreover, as Armstrong notes, registration of vital statistics led to greater specificity in analysis of death: ‘the notion of a pathological cause of death in the form of a disease was introduced to replace so-called “natural” death’.^36^ Doctors came under ever greater pressure to frame the specific cause of death in clinico-pathological terms in all cases.^37^

In the absence of an obvious cause, sudden infant deaths were better suited to a system which simply recorded the fact of death. With growing emphasis on providing a specific clinico-pathological cause, one of two courses could be followed. The death might be registered under an alternate heading—such as accidental suffocation or respiratory disease. This practice entailed that the scale of sudden infant death was not recognised until studies began to examine the problem in more depth in the middle decades of the twentieth century. Alternatively, the case could be referred to a coroner for further investigation.^38^ While coroners were often legally trained, their investigation might draw on the expertise of a doctor specialising in forensic medicine or a pathologist's autopsy report.^39^ However, this was rare in cases of sudden death in infants. One London coroner suggested that county councils objected to the expense involved.^40^ Another pointed to procedural reasons:

It may be that a coroner will act on the strength of the doctor's opinion as to the cause of death without necessarily having the confirmation of a post-mortem examination. Prior to 1927 this was usually the case, as it was not then possible for a coroner to order a post-mortem examination without holding an inquest; therefore the medical opinion as to the cause of death, based on circumstantial evidence and external evidence only, was usually accepted.^41^

The *Coroners (Amendment) Act*, 1926, introduced scope for a coroner to request an autopsy without automatically provoking an inquest, provided the death was found to result from natural causes. This gave pathologists unprecedented opportunity to examine the accidental suffocation hypothesis frequently recorded as the cause of sudden infant death. Subsequently, local studies reported findings from increased numbers of post-mortem examinations raising doubts about the validity of the overlaying, or accidental suffocation, hypothesis.^42^ These doubts, brought to the attention of the Ministry of Health in the 1950s, were the proximate cause of the coordinated studies on sudden infant death that took place in the 1950s and 1960s.

Evidently, it is important to understand the rise of medical interest in sudden infant deaths, and the historicity of SIDS as a diagnosis, in relation to developments in the registration of vital statistics, medical law and paediatrics in the late nineteenth and early twentieth centuries. While these factors combined to produce a context conducive to the medicalisation of sudden infant deaths by the 1960s, there had been earlier attempts to provide medical explanation and intervention for sudden infant deaths.^43^ Ann Dally's work on Status Lymphaticus reveals that up until the 1950s, British medical students were taught that an enlarged thymus gland could indicate susceptibility to sudden death in infants, children and adults. In some versions of the theory, the enlarged gland itself was the direct cause of death—inhibiting circulation and respiration in the victim. By other accounts, an enlarged thymus was indicative of a constitutional weakness that predisposed the individual to unexpected sudden death from apparently trivial causes, such as the administration of inoculations in childhood or chloroform during surgery.^44^

Uncertainty regarding the function of the thymus gland contributed to medical advice that, in cases where it was enlarged, it should be removed. However, there was no established norm regarding the size of the thymus. As Dally details, the decision to establish a norm using the findings of autopsies on children who had died in hospital had disastrous consequences, as it was subsequently revealed that this cohort presented with atypically small glands as a consequence of the role of the thymus in combating infection. Normal glands were categorised as dangerously large—an error compounded by the negative effects of advice to surgically remove the thymus, or use irradiation to reduce its size. Surgical interventions had high mortality rates, and irradiation was subsequently linked to high rates of cancer. An inaccurate understanding of the aetiology entailed that measures intended to be prophylactic caused significant iatrogenic harm to healthy individuals. Dally's research details specific medical interest in sudden infant death during the first half of the twentieth century, highlighting the considerable risks involved in attempting to identify a causal mechanism and develop prophylactic interventions. While an MRC committee report in 1931 concluded that there was no such thing as Status Lymphaticus, the theory continued to find support into the 1940s.^45^

In summary, by the mid-twentieth century there was increased medico-legal focus on infants, and on identifying and recording the specific causes of infant mortality, framed in clinico-pathological terms. Changes to the *Coroners Act* in 1926 provided greater scope for investigation of cases where the specific cause of death was unclear, allowing pathologists to test the evidence for existing theories regarding ‘overlaying’ and accidental suffocation as the causes of sudden infant death. But the early decades of the twentieth century also highlighted the risks of prophylactic medical intervention. Against this backdrop, the Ministry of Health began to support research into the aetiology of sudden infant deaths in the 1950s.

## Ministry of Health and MRC Studies

In 1951, Professor Samuel Bedson, renowned bacteriologist, and Dr Francis E. Camps, forensic pathologist, wrote a memorandum to the Ministry of Health.^46^ They suggested that there was growing evidence that some cases of sudden death of infants attributed to accidental mechanical suffocation might be due to other causes, such as acute infection.^47^ The Ministry of Health invited Leslie Banks, Professor of Human Ecology at Cambridge, to chair a steering committee to investigate further.^48^ In particular, the enquiry was targeted towards determining whether the deaths often attributed to accidental suffocation were actually a result of bacterial or viral infections, or the inhalation of food or vomit. A preliminary study confirmed the weakness of the accidental suffocation hypothesis, and also suggested that the problem was larger than previously acknowledged—1,400 cases annually in England and Wales, equating to over 20 per cent of postneonatal infant mortality.^49^

Banks' committee conducted the enquiry under three broad headings, examining microbiological, pathological and social factors. The investigation was concentrated in Cambridgeshire, where previous investigation had taken place, and the London County Council area.^50^ On receiving notification of the sudden death of a child, aged between two weeks and two years old and living in one of the study areas, coroners were to report the findings of their investigation on a form designed to facilitate the study. Part one of the form recorded post-mortem findings, including results of histological and bacteriological tests.^51^ The second section recorded social data and was completed by a health visitor assigned to investigate the background and circumstances of death. This covered prior signs of illness or symptoms, the location and position in which the body was found and any other findings relevant to evaluating suffocation as the likely cause of death.

In 1958, the interim report of the committee identified trends in the 81 cases investigated.^52^ Sudden death was most common in the first year, affected boys more than girls and peaked in winter. The report underlined the need for more detailed pathological investigation. While naked eye pathological appearances might suggest mechanical asphyxia, some thought histological findings indicated acute respiratory infection.^53^ However, few deaths could be ascribed to bacterial infection and early results appeared to exclude this as an important factor.^54^ Consequently, bacteriological analysis of cases was discontinued in London. It continued in Cambridge, although a further 50 cases also produced negative findings.^55^ Similarly, evidence of a virus was isolated in only two of the 50 London cases in which testing had been undertaken. In three other cases the presence of a virus could not be ruled out.

Analysis of the social factors cast further doubt on the overlaying/accidental suffocation hypothesis. Only nine cases were recorded as occurring when the infant was in bed with a parent and there was little evidence for other causes of accidental suffocation. The previous standard of care for victims was recorded as good, and while in many cases parents recollected a history of minor symptoms, such as a slight cold or some vomiting of feeds, ‘these were almost all of an apparently trivial kind’.^56^ As was noted at the time, such recollection after death could be ascribed to the obvious desire to find any sort of foothold for investigation and explanation.^57^ While the interim report noted that the study had not yet identified the cause of sudden death, after an MRC review of the interim findings it was agreed that the work should continue. Although recognising that the social investigation was important, the report concluded that the intensification of virus investigation was the crux.^58^

The MRC review had been undertaken by four referees. Sir Wilson Jameson, Sir Wilfrid Sheldon and Dr Alan Carruth Stevenson were in favour of continuing the work.^59^ By contrast, Sir Dugald Baird was unimpressed by the interim report and doubted that there was sufficient value in continuing the research.^60^ Such doubts were not reflected in the reply sent to the Ministry of Health:

The concensus (sic) of opinion of those referees we have consulted is that there is a need for studies of this kind, and in view of the promising start that has been made it would seem appropriate for Professor Banks and his colleagues, should they so wish, to submit concrete proposals for future work for consideration by the Board.^61^

This offer of funding from the MRC Clinical Research Board (CRB) was accepted in order to add immunology to the lines of investigation. In 1960, a team of researchers from Cambridge and London published research demonstrating that inhalation of cow's milk could produce a severe immunological reaction leading to sudden death in sensitised guinea pigs.^62^ They believed this might provide an explanation of cases of sudden death involving artificially fed infants and MRC funding was used to explore the hypothesis. Mavis Gunther had been investigating the effects of bottle feeding on infants, and, combining this with Robin Coombs' work in immunology, a collaborative project was devised to assess whether an overwhelming immunological reaction might result from regurgitated milk getting into the respiratory tract or lungs of the infant.^63^ The significance of extending the Steering Committee's study in this direction was twofold. First, immunological work became the major focus of British research into sudden infant deaths. Secondly, Coombs became a critic of the Ministry of Health Steering Committee, and a catalyst for the promotion of MRC research.

Prior to departing for Washington to attend the first international symposium on the causes of sudden death in infants, in July 1963 Coombs wrote a letter urging the MRC to set up a small working party or conference to discuss cot deaths.^64^ The letter was partially motivated by the fact that his recent work with Parish was coming to an end. He felt that an independent evaluation of the research was necessary before developing it further with Gunther, who had a funding application pending at the MRC. Although their experimental findings had yet to produce convincing evidence in support of the anaphylactic shock hypothesis, there were still promising leads. However, ‘the next move is a rather difficult one, and all depends on how much credence one puts on the hypothesis in the light of all our experimental evidence.’^65^

Evidently they were struggling to prove that a modified anaphylactic reaction, triggered by the regurgitation of cows' milk into the respiratory tract or lungs of a sensitised sleeping infant, was a likely cause of cot death. While there was some evidence for this hypothesis in results of their tests in anaesthetised guinea pigs, it was no more than circumstantial. Nonetheless, even without solid proof that anaphylaxis resulting from hypersensitivity to cows' milk was definitely the cause of some cases of cot death, they might still assist in reducing the risk of such deaths. Their strategy focused on infant feeding, in particular the risks associated with the use of dried milk powders specially treated to render the soluble proteins insoluble in the stomach. If ways could be found to reduce the likelihood of infants regurgitating artificial feeds, then the risk of cot death resulting from anaphylactic shock might be correspondingly reduced regardless of the fact that the specific mechanism or aetiology had not been experimentally proven.

The MRC extended Gunther's funding and this research later informed the recommendations on breastfeeding and research on artificial feeds arising out of the Steering Committee's final report.^66^ On his return from Washington, Coombs arranged a meeting to discuss the possibility of an MRC conference. Evidently he was not inspired by the current work of the Steering Committee. He suggested that it was ‘a committee of amateurs’ in need of expert guidance and opinions. As it had not met for two years, he regarded it as ‘moribund’ and indicated that its chairman was aware, and approved, of his request that the MRC set up another body.^67^ Negotiations towards arranging a small conference took place over subsequent months. While there was Ministry of Health support for an MRC sponsored meeting, this was delayed until publication of the final report of the Steering Committee.

The Steering Committee issued its final findings in autumn 1964. Following the recommendation of its interim report, exhaustive virological examination had been undertaken on specimens from a further 51 cases but results remained negative. Consequently, this aspect of the work was suspended in 1961 in order to concentrate on analysis of sociological and immunological factors. Investigation of social factors was refined, omitting questions that prior analysis had shown to be irrelevant and adding more detailed questions about bedding, the clinical history of cases and the use of medicines.^68^ It was also extended to incorporate control group data, undertaking the same questionnaire with parents of healthy children of the same age and sex, and drawn from the same residential areas, as cases of sudden death.

Findings from each strand of investigation were used to place cases into one of two categories: ‘explained’ or ‘unexplained’. The former included cases in which either a definite or a possible cause of death had been identified.^69^ The ‘unexplained’ category was reserved for cases where detailed investigation resulted in no cause being identified. Out of a total of 130 cases in which the history and macroscopic investigations were backed up with detailed histological post-mortem investigations, 93 were classified as ‘unexplained’. Both the bacteriological and viral investigations echoed the negative findings of the interim report. The analysis of social circumstances drew on reports of 152 cases, of which 108 remained unexplained. There was increased recollection of symptoms of respiratory illness from the parents of sudden death cases when compared to the control group—68 per cent compared to 31 per cent. Echoing the interim findings, these were often very trivial—snuffles or a slight head cold—and had not given cause for anxiety at the time. The final report acknowledged the likelihood of bias, suggesting that at least some of the difference could be attributed to the fact that parents of victims ‘seeking to find some explanation for the tragedy, are likely to recall any little symptom, whereas the mother of a healthy lively control child might easily forget that the baby had a slight cold ten days previously.’^70^ A short period of breastfeeding and early bottle feeding; the use of a soft pillow; illegitimacy; being born to a young mother; low household income; low social class; and poorer quality housing were all identified as factors leading to an increased risk of sudden infant death. Clearly then, while cases of sudden infant death were found across the socioeconomic spectrum, the risk factors identified for sudden infant death echoed earlier concerns surrounding overlaying—highlighting poor families, living in bad housing, and illegitimacy as major factors.^71^

The immunological research had focused on establishing evidence that hypersensitivity to cows' milk might provoke fatal anaphylactic shock. Studies of lung tissue were largely inconclusive. Similarly, evidence of cows' milk in the lungs of 25 out of 60 ‘unexplained’ cases was noted, although this finding was contextualised by the fact that it was also present in 8 out of 25 ‘explained’ cases—suggesting that it might be a characteristic, rather than a trigger, of terminal events. Perhaps most significantly, serum antibody titres to cows' milk protein were on average higher for ‘unexplained’ cases than for normal infants of the same age. Combined with the fact that ‘unexplained’ cases were more likely to be bottle-fed, this aspect seemed worthy of further investigation.

Although a large number of deaths remained unexplained after detailed investigation, the report concluded by highlighting what appeared to be statistically significant risk factors: early bottle-feeding, the use of a soft pillow, and, at least in some cases, recent infection. Therefore, the report recommended that certain precautions be taken. If a pillow was used for sleeping, it should be hard rather than soft. All babies should be exclusively breastfed for the first two weeks of life. In order to minimise any risk of fatal anaphylactic shock resulting from inhalation of cow's milk, a means of treating cows' milk should be found to ensure that all the proteins coagulated in the infant's stomach, thereby minimising the risk of regurgitated food proteins provoking an overwhelming immunological response.^72^

Having taken the Steering Group's investigations as far as he felt possible, Banks suggested to Sir George Godber that the MRC should determine if further investigation was necessary.^73^ Godber and Sir Harold Himsworth decided that any future meeting should be confined to consideration of further research on the anaphylactic shock hypothesis.^74^ An MRC working party conference was convened, under the Chairmanship of Professor Arnold Ashley Miles on 16 June 1965.^75^ Coombs, who had originally pushed for the meeting, was asked to present a paper on the anaphylactic hypothesis as a starting point for the discussion. Echoing his previous comments to the MRC in 1963, Coombs' presentation noted the strengths and weaknesses of attempts to produce firm evidence for the theory. In the ensuing debate, the participants expressed reservations, pointing to the circumstantial, unconvincing nature of the evidence produced to support the hypothesis, highlighting in particular the problem of using anaesthesia to mimic sleep conditions in guinea pigs.

In summing up, Miles felt that no clear programme of further investigation had emerged from the meeting. There was consensus of opinion that, despite equivocal evidence for the anaphylaxis hypothesis, some further work should be undertaken—a conclusion driven more by a general interest in further research on infant immunology than any clear belief that it was likely to reveal the aetiology of unexplained infant deaths.^76^ The recommendation passed on to the MRC was that, if its CRB was in favour of pursuing research, a working party should be set up to examine the validity and feasibility of further work on the cows' milk protein hypersensitivity hypothesis.^77^ In fact, the CRB advised that such a working party was unlikely to serve any useful purpose. More generally, there was little evidence of desire to undertake significant further research on this aspect. Coombs received a small grant, but when the MRC approached other researchers there was reluctance to develop work on the anaphylaxis hypothesis.^78^ Even Gunther, whose research had been supported by ongoing grants from the MRC, declined the opportunity to apply for funding to develop a new project. She cited the need to concentrate on writing a book on infant feeding which had been set aside whilst working on cot death research:

the ending of my grant should be seen quite apart from the need for a structure of some sort to consider further work on cot death. The events have been like a game of chess with a sequence of moves. The Council took over formally from the Ministry's Steering Committee and then by limiting all future consideration to aspects of milk allergy and then finding no means of tackling milk allergy, have cornered the subject into absolute immobility.^79^

Gunther urged the MRC to set up a new committee to consider various lines of enquiry, including the virology of intrauterine and neonatal infections; further study of the peculiarities of the immune state in the first six months of life; more detailed post-mortem histology of cot death; and further statistical analysis of the registration of cases. She enclosed a copy of an article she had recently written, which postulated that exposure to viruses, such as rubella, could result in intrauterine or neonatal infection and the development of antibodies. As an alternative to the artificial feeding hypothesis, Gunther suggested that a severe antibody-antigen reaction may be another possible cause of anaphylactic shock resulting in cot death.^80^

Replying the following month, the MRC indicated that a new committee was unlikely. The letter stressed that they had not taken over investigation of sudden infant deaths from the Ministry of Health, but rather, in discussion they had come to the view that further examination of the hypersensitivity hypothesis would be a productive next step. However, they had been unable to stimulate interest in undertaking new work in the field.^81^ Nonetheless, the letter expressed a desire to encourage research on the virology of intrauterine and neonatal infections. To this end, the MRC gauged expert opinion on the merits of Gunther's idea. The respondents were in favour of investigations into the role of viruses in cot death, in light of recent advances in knowledge of respiratory viral infections, although they recognised a potential difficulty in obtaining suitable material for study. Dudgeon, who already had MRC funding for research on rubella, suggested he could follow this up as an aspect of his current work. He indicated that his colleague, Dr Soothill, an immunologist, might also be interested in undertaking some investigation.^82^

## The Second International Conference, 1969

‘The first step towards the solution of any problem is the recognition of the existence of that problem’ was Marie Valdes-Dapena's observation whilst summarizing progress between the first and second international conferences on sudden infant death.^83^ The conference in 1963 had recognised the existence of the problem and since then work had begun on finding a solution, and, drawing on her earlier published review of the world literature on sudden infant deaths, Valdes-Dapena summarised the findings of recent research.^84^ This echoed both the range of hypotheses examined in Britain and also the negative results of attempts to establish a causal explanation. Whilst acknowledging British support for the anaphylactic shock hypothesis linked with hypersensitivity to cow's milk, Valdes-Dapena noted that attempts to evidence the theory had been unsuccessful.^85^ She also highlighted Gunther's paper on intrauterine infection as a possible source for a hypersensitivity reaction. While noting that the theory had merit, the fact that studies had not consistently isolated evidence of viral infection was a ‘major stumbling block’.^86^

Valdes-Dapena's presentation was followed by a discussion of terminology, led by Bergman and Beckwith, which resulted in the decision to use Sudden Infant Death Syndrome.^87^ The new diagnostic term was deemed to have ‘the important virtue of communicating to the medical profession the concept that this is, in fact, a distinctive clinico-pathological entity’ which made it preferable to the potentially misleading ‘cot’ or ‘crib’ death.^88^ However, despite strong rhetoric emphasising the strides taken towards confirming SIDS as a ‘real disease’, studies had yet to identify a clear aetiology, or even signs that could be used to identify cases at risk prior to death.^89^ SIDS was a disease without known symptoms, signs, explanatory pathology or patients. This highlights a further factor in the historicity of SIDS: the medicalisation of such deaths required an ontological model of disease.

## A Disease without Symptoms, Signs, Pathology or Patients

In his introductory chapter to *Framing Disease*, Charles Rosenberg highlights that the physical manifestation and perception of symptoms is the starting point of defining disease.^90^ However, the importance of symptoms in the diagnostic process has changed over time. The patient's narrative case history, prominent in eighteenth-century medicine, was gradually absorbed into models of disease based on standardised understandings of pathological anatomy, supplemented by germ theory and the growing importance of laboratory based analysis.^91^ Technology increasingly facilitated the diagnostic process, with implications both for the diagnosis of disease before symptoms became manifest to seemingly healthy individuals, and for those unable to effectively communicate their own case history. Increasingly, the patient's awareness and experience of disease and the scientifically defined existence of disease could be separated.

As Rosenberg notes, the ‘modern history of diagnosis is inextricably related to disease specificity, to the notion that diseases can and should be thought of as entities existing outside the unique manifestations of illness in particular men and women’.^92^ This formulation is one example of an idea expressed by a number of historians of medicine relating to a shift from a physiological concept of disease to an ontological concept of disease.^93^ The former understood disease as a general constitutional imbalance, particular to, and largely inseparable from, its manifestation in individual patients. The latter understood disease as a localised and standardised entity which could be more easily theorised and analysed apart from its presence in specific patients. The ontological idea of disease as specific entity was connected with the rise of scientific and laboratory analysis as the basis of diagnostic medicine over the course of the nineteenth and twentieth centuries.^94^

Writing in the 1970s, when SIDS was becoming established as an internationally recognised diagnosis, Stanley Reiser argued that medicine increasingly prioritised the importance of objective evidence provided by scientific laboratory tests and technological investigation of the body, over patients' subjective accounts of their symptoms or doctors' unaided observations.^95^ Aside from the obvious point that infants were unable to describe their case history, victims of sudden unexpected death were, by definition, unlikely to have been active patients providing subjective evidence of illness to attract medical attention prior to death. A SIDS diagnosis could only be retrospectively applied to the deceased following autopsy. The growth of the ontological concept of disease allowed for analysis in isolation from patient experience. This gave greater scope for the medicalisation of sudden infant deaths as SIDS, a disease diagnosis, in the second half of the twentieth century. However, it was still a jump to get from a more amenable context for the recognition of sudden infant death as a medical problem, to the acceptance of SIDS as an official diagnosis. For, as Canguilhem notes, the development of a standardised model of disease, potentially allowing it to be diagnosed even before symptoms have become manifest to the patient, is typically a result of the recording and investigation of previous cases.^96^ With SIDS, the absence of symptoms, signs or explanatory pathology in previous cases made it difficult to develop a standardised model that could be used to diagnose potential cases of SIDS in living infants.

**Fig. 1:**
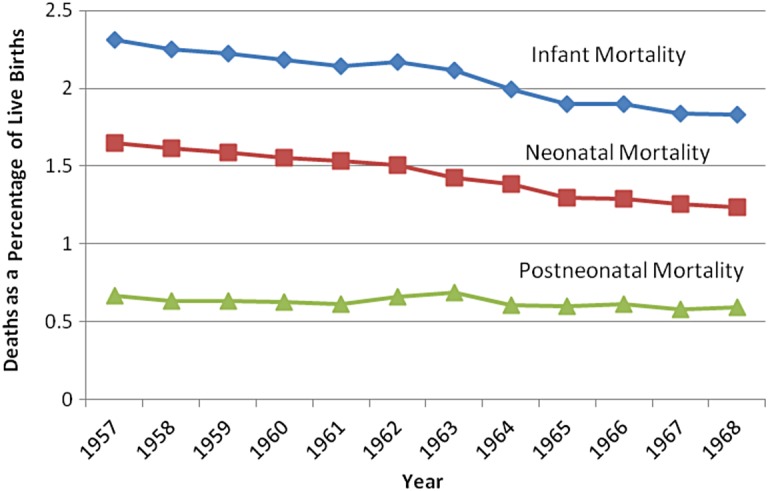
Infant mortality rates in England and Wales, 1957–1968

*Source*: Based on the figures contained in Table 1.2 in the Department of Health and Social Security, *Confidential Enquiry into Postneonatal Deaths 1964–1966* (London: HMSO, 1970), 4.

An alternative option was to use epidemiology to seek an objective understanding of the disease derived from analysis of population level statistics rather than the symptoms or signs from individual patients. As Figure 1 demonstrates, in the decade prior to the definition of SIDS, overall infant mortality was decreasing, primarily as a result of falling neonatal mortality. Although numerically smaller, postneonatal mortality was relatively static over the period.^97^

As the Department of Health and Social Security's enquiry made clear, the comparative trends made postneonatal mortality a significant cause for concern, provoking further investigation.^98^ The enquiry highlighted that 37 per cent of postneonatal mortality was attributed to ‘cot death’, and later studies drew attention to such deaths as the largest individual cause of mortality in this group.^99^ The increasing prominence of sudden infant deaths attracted medical attention—a point noted in Emery's writings on the subject.^100^

However, when considered at the level of population statistics, these deaths posed problems for epidemiologists. Peter Froggatt identified three difficulties: definition, post-mortem diagnosis and controls.^101^ Death registration forms used categories from the International Classification of Diseases which provided a universal set of commonly understood diagnoses intended to produce uniform and comparable statistics across regions and countries. However, there were grey areas in classifications and inconsistency in registration practice.^102^ Sudden infant deaths might be registered under a variety of headings, reflecting differences in local understanding and approach, and depending on whether a full case history had been taken and/or an autopsy carried out. Echoing findings of earlier studies, Froggatt noted that the registered cause of death could be influenced by the socioeconomic circumstances of cases. Suspicion of neglect or foul play in cases from poorer backgrounds could lead to a registration of ‘accidental suffocation’ or ‘cause unknown’. In more affluent areas, doctors might register a clear cause, even in the absence of evidence, in order to ease the upset and avert any stigma of suspicion. Cases of sudden infant death shared characteristics with respiratory deaths—a point evidenced by the subsequent diagnostic transfer from respiratory infection to SIDS in the 1970s and 1980s—and the former were often registered as the latter despite the absence of any evidence of respiratory infection. Inconsistent registration inhibited the development of longitudinal trends in sudden infant death, affecting comparisons of incidence across geographical regions.

Arguably, SIDS was adopted as a diagnosis because it would facilitate better registration statistics. Given his reservations about the lack of positive diagnostic criteria, Emery favoured SIDS being entered as secondary information after whatever the pathologist considered was the most likely primary cause.^103^ Certainly, increased medical awareness of the issue impacted on registration figures. Between 1971 and 1988 the postneonatal mortality rate in England and Wales dropped from 5.9 to 4.1 per 1,000 live births, while the recorded rate of SIDS increased from 0.3 to 2.0 per 1,000 live births—reflecting an increase from 5 per cent to nearly 49 per cent of overall postneonatal mortality.^104^ The increase in SIDS mirrored a corresponding decline in deaths from respiratory conditions, indicating diagnostic transfer. Between 1988 and 1992, there was sharp decline in all forms of postneonatal mortality, including SIDS.^105^ Rates were stable between 1992 and 1997, before transfer away from SIDS to other causes of death. Recent studies indicate that, while the term ‘SIDS’ is still used, pathologists register cases under a variety of terms—SIDS; SUDI; ‘unascertained’—reflecting local preferences and carrying nuances of interpretation.^106^

Froggatt's second and third problems were the fact that SIDS was solely a post-mortem diagnosis and it was difficult to identify suitable controls for comparative study. This limited the range of potential investigations and made it difficult to determine whether risk factors identified for the SIDS group were characteristic of SIDS in particular.

The resultant study population will be biased toward factors known to be associated with infant mortality in general—that is, in all probability, toward bigger and poorer families, a preponderance of males and of winter deaths, a lower birth weight, and so on. We note, of course, that these are the very factors incriminated in sudden unexpected death.^107^

While epidemiological research highlighted sudden infant deaths as an area of concern, this was only a tentative step towards identification of a disease. Progress required the development of causal explanations demonstrating the mechanism(s) by which the risk factors resulted in sudden death, followed by experimental testing of the hypotheses. However, as Dally's account of medical interest in Status Lymphaticus underlines, there were significant risks in attempting this using healthy infants; and the work of Coombs *et al.* illustrated the difficulty of using animal models.^108^

Emery's publications highlight the importance of statistics to the rise of medical interest in SIDS, but without symptoms, signs or pathology, it is obvious why he suggested SIDS appeared to be a diagnostic dustbin. However, the validity of this provocative label is dependent on regarding diagnoses purely in terms of symptoms, signs and pathology. Rosenberg notes that the act of diagnosis not only structures medical practice but also confers social approval on particular sickness roles and legitimises bureaucratic relationships.^109^ Conceptualising particular characteristics as a disease both reflects and shapes social attitudes. As Elizabeth Fee puts it, ‘medicine itself is not neutral but carries both liberating and repressive functions’.^110^ A medical diagnosis can influence society's judgement of individuals in positive or negative ways. In cases of SIDS, the diagnosis provided a medical model of understanding that presented the first step towards an explanation, helping to counter legal suspicions of intentional harm that often surrounded the parents of SIDS victims.

Many publications open with personal accounts of cases, paying tribute to the role that the families of SIDS victims have played in promoting medical interest and research.^111^ Bergman notes the death of Mark Addison Roe in Connecticut in 1958 was a catalyst for scientific research into SIDS in the USA. Having been pronounced healthy at a check-up two weeks beforehand, Mark's sudden death left his parents seeking an explanation. Unable to identify a suitable foundation or research project, in 1962 the Roes decided to set one up using the substantial insurance policy Mark's grandfather had taken out at his birth.^112^ Thus, for Bergman, in America the change in medicine's attitude to SIDS came about ‘not through any actions of the high priests of science and medicine, nor through the initiatives of government policy makers. Change was wrought by a small band of parents who had lost babies to SIDS, aided by a few good-hearted physicians by means of an organised political campaign.’^113^

In Britain, the Foundation for the Study of Infant Deaths was established in 1971 at a symposium, held in Cambridge, which arose out of a grandmother's attempts to find an explanation for her infant grandson's unexpected death.^114^ During this meeting, Professor Wedgwood, of the department of pediatrics in Seattle, presented some work of his colleagues, Bergman and Beckwith, which made clear their awareness of the significance of providing a medical diagnosis:

The result of the tragedy is not only the loss of life of an infant, but also the disruption of the family as a personal and social unit. The incident has profound psychological and social concomitants. Thus recognition and understanding both of the biology and the sociology of the syndrome, and hopefully in the future the prevention of the deaths has importance that goes far beyond the immediate death of the single infant—it reflects on the whole well-being of a family. Only by recognition and understanding of *both* these facts—the occurrence of the syndrome and the prevalence of the family reactions—can the physician assist properly in his chosen role. As scientists we may be more comfortable in discussing and studying the biological process; but we are negligent if we disregard the psychological and social implications of this disease.^115^

Where typically a medical diagnosis was of great significance to the patient diagnosed, in the case of SIDS there were no patients. Rather, the diagnosis was only of value to others—paediatricians, pathologists, epidemiologists, medical researchers and, above all, to the parents and relatives of victims. It deflected some of the legal suspicion falling on parents and provided the beginnings of an explanation for the loss of their child. It became a focus for the families of victims, who organised themselves into support groups and raised funds to support research.^116^

So while SIDS may lack positive criteria or prophylactic and therapeutic value, it would be wrong to regard it as simply a diagnostic dustbin. As Emery noted, the diagnosis has great significance for parents who can be assuaged of guilt that they did something wrong or failed to do something right,and for doctors—who may be concerned that they missed some sign of disease.^117^ It also legitimates the phenomena as an area of medical research.^118^

However, the medicalisation of sudden infant deaths did not entirely remove legal suspicion of infanticide, especially when repeat cases arose within the same family. Without clear aetiology, positive diagnostic criteria or pathological evidence, sudden infant death remained a penumbral area of law, where the opinion of expert medical witnesses was often pivotal in deciding cases. In some instances where expert opinion convicted mothers accused of killing their infant(s), the evidence was subsequently discredited and the convictions overturned—the deaths being attributed to other causes, including SIDS.^119^ Several such high profile cases led the Attorney General to order a review in 2004.^120^

## Conclusion

In the case of Britain, SIDS was not an ignored disease. Rather, analysis points to the importance of recognising the historicity of SIDS—a diagnosis facilitated by the confluence of changes in medicine and law towards the middle of the twentieth century. The decline in other categories of infant mortality meant that sudden infant deaths became an increasingly prominent concern. Legislation facilitated greater numbers of autopsies, giving unprecedented opportunities to test theories of overlaying. And, the growing emphasis on ontological concepts of disease meant that it was easier to medicalise such deaths despite the absence of a patient. In sharp contrast to Bergman's account of the challenges faced in gaining federal funding for SIDS research in the USA, it is clear that, in Britain, there was early support from both the Ministry of Health and the MRC.^121^ Indeed, by the mid-1960s the MRC had difficulty persuading researchers to undertake further immunological investigations.

However, the growth of medical interest and the use of “SIDS” did substantially impact on registration practices. The large-scale diagnostic transfer to SIDS underlines the importance of a diagnostic label in bureaucratic medicine, and might be regarded as significant in revealing the extent of the problem. However, as Froggat noted, the risk factors for SIDS were characteristic of infant mortality generally, so the category would have been an appealing catchall for many cases. Over time, the flow was reversed as advances in technology and knowledge facilitated the identification of an alternate specific cause for some cases, and uncertainty over SIDS as a diagnosis led some pathologists to register deaths under other headings.

This does not mean that SIDS was nothing more than a diagnostic dustbin. As noted in later sections, although the term had little therapeutic or prophylactic value, it had great significance in other respects. It could reduce the suspicion cast on parents, and alleviate some of the guilt stemming from fears that they had done something wrong or failed to do something right. In that sense it had great psychological and socio-legal value. As such, it illustrates the growing authority and status of medicine—producing a medical model of explanation that encroached into the traditional legal frameworks of understanding and response. At a time when critiques were bringing to light cases of iatrogenic harm, abuses of medical power and the dangers of overly authoritarian and paternalistic approaches to medical research,^122^ SIDS provides an example of more beneficent medicalisation of life and death. The relatives of victims welcomed a medical diagnosis which could provide them with a starting point towards explanation of the sudden death of their infant; could help assuage concerns or guilt that a symptom or sign had been missed; and could counter legal suspicion of infanticide.

SIDS was neither an ignored disease nor simply a diagnostic dustbin. To medical historians it presents a valuable case study of both the historicity of disease and the numerous ways in which it can be identified, defined and understood. Above all, SIDS provides a unique example of an officially recognised disease that, by definition, had no patients, and in which the certainty of the diagnosis was greatest whenever there was least evidence of symptoms, signs or pathology.

## Funding

This work was supported by the Wellcome Trust as part of an Enhancement Grant to the Centre for the History of Medicine at the University of Glasgow. Grant number 074301/Z/04/Z/AW/HH.
